# The Snakin Family of Antimicrobial Peptides: Promising Alternatives to Conventional Antibiotics

**DOI:** 10.3390/ph19050788

**Published:** 2026-05-18

**Authors:** Tuğba Teker, Gülruh Albayrak

**Affiliations:** 1 Department of Molecular Biology and Genetics, Faculty of Engineering and Natural Sciences, Istanbul Atlas University, Kağıthane, Istanbul 34408, Türkiye; 2 Department of Molecular Biology and Genetics, Faculty of Sciences, Istanbul University, Vezneciler, Istanbul 34134, Türkiye; gulruh@istanbul.edu.tr

**Keywords:** antimicrobial peptide, plant-derived AMPs, antimicrobial resistance, snakins, antibiotic alternatives

## Abstract

Antibiotic resistance has become a significant challenge for global health. Exploring novel antimicrobial compounds as alternatives to antibiotics is increasingly prominent in combating resistant pathogens. Antimicrobial peptides (AMPs), produced by various organisms, are considered natural antibiotic candidates that can be used against multidrug-resistant microorganisms. The snakin family of plant-based AMPs is a promising candidate for use in the agriculture, food and pharmaceutical industries due to its antimicrobial activity against both phytopathogenic and clinical species. This review summarizes current AMP databases and the snakin family of plant AMPs deposited in the Universal Protein Resource, UniProt. It also provides knowledge about potential uses of this family in biotechnology.

## 1. Introduction

Antimicrobial peptides (AMPs) are innate immune response oligo- and/or polypeptides consisting of amino acids ranging from 10 to 100 in number. They are synthesized by bacteria, archaea, and various eukaryotic organism groups in response to continuous or biotic and/or abiotic stimuli in the metabolic process [1,2]. AMPs are more abundant in specific organs, tissues, and cell types exposed to a wide range of pathogens, including viruses and parasite, leading researchers to suggest that they constitute the first line of innate immune defense [3,4,5]. They can impede the development of pathogenic infections before symptoms occur or participate in the inflammatory response of the host organism [1].

**Figure 1:**
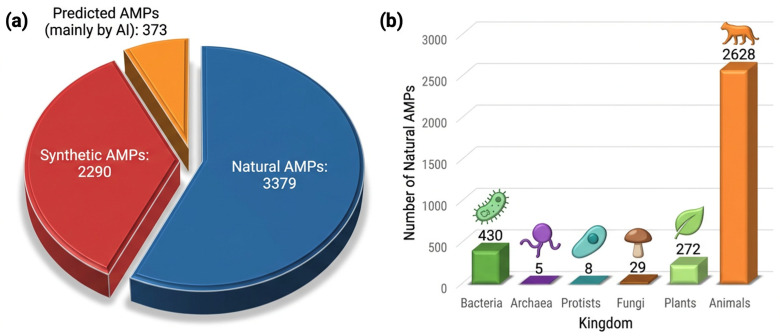
Distribution of antimicrobial peptides (AMPs) registered in the APD6 according to their types (**a**) and natural AMPs across six kingdoms (**b**). The figure was created using FigureLabs (AI Agent for Scientific Illustration) according to the Antimicrobial Peptide Database data [6].

The first discovered AMP is gramicidin, isolated from soil-borne *Bacillus brevis* by Dubos in 1939 [1,2]. To date, more than 6000 characterized AMPs have been deposited in the Antimicrobial Peptide Database (APD). Currently, the APD comprises 6309 peptides, including 3379 natural AMPs (~53.6%) and 2290 synthetic AMPs (~36.3%). In addition, 373 predicted AMPs via artificial intelligence (~5.9%) are contained in this database [6] (Figure 1a,b). They are classified based on different parameters such as origin, structure, physicochemical properties and mechanisms of action, biological activity, and biosynthesis [2,7,8]. This information, such as properties and biological activities, can be accessed from various databases, some of which are listed in Table 1.

**Figure 2:**
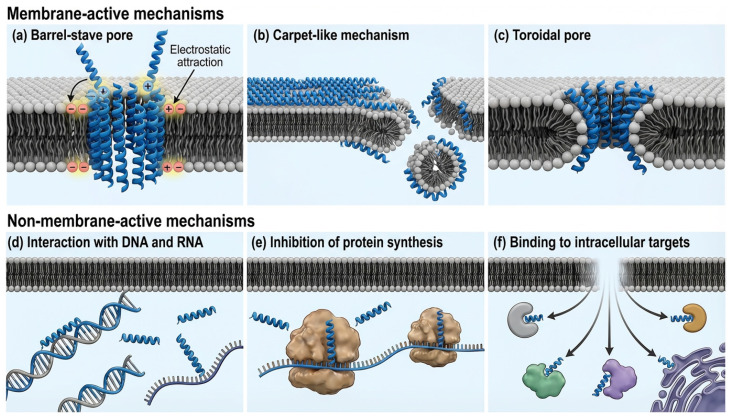
Schematic representation of membrane-active (**a**–**c**) and non-membrane-active (**d**–**f**) mechanisms of AMPs acting on the membrane model. The figure was created using FigureLabs (AI Agent for Scientific Illustration).

AMPs have been demonstrated to promote the immune response by inducing the recruitment of immune cells responsible for pathogen elimination and/or inhibition of inflammation [1,7]. Researchers have focused on the interaction of peptides with model membrane systems using artificial lipid membranes, rather than membrane potential sensitive dyes or fluorescent labeling approaches, in studies on the action mechanisms of cationic AMPs in microbial systems. The results demonstrated that these cationic AMPs initially interact with microbial membranes through electrostatic interactions; however, their mechanisms of action occur in two ways: membrane-active (membrane-targeted, membrane-disruptive) and non-membrane-active (non-membrane-targeted, non-membrane-disruptive) [42,43]. The first target of membrane-active peptides is the anionic phospholipid layer of the cell membrane. They accumulate on the surface of the membrane through electrostatic and hydrophobic interactions. Then, they bind to the phospholipid layer and disrupt the membrane integrity by various mechanisms. They disrupt membrane integrity by forming a pore structure in the membrane in the barrel-stave and toroidal pore models, while they disrupt it by forming a micelle structure with membrane components in the carpet-like model [44,45]. In the barrel-stave model, AMPs insert perpendicularly into the lipid bilayer and generate the transmembrane pore (Figure 2a). In the carpet-like model, they bind parallel to the membrane surface via electrostatic interactions between cationic peptides and negatively charged phospholipid head groups. Upon reaching a critical concentration, they reorient and move toward the interior of the membrane, leading to micelle formation and membrane pore (Figure 2b). In the toroidal pore model, AMPs insert perpendicularly into the lipid bilayer. This arrangement induces local membrane curvature by pushing apart phospholipid head groups, resulting in a pore lined by both AMPs and lipid polar head groups leading to the formation of toroidal pore, unlike barrel-stave pores (Figure 2c).

AMPs can also display their effects as non-membrane-targeted approaches by directly crossing bacterial membranes via energy-independent and energy-dependent mechanisms, thereby reaching intracellular targets [46]. They inhibit DNA, RNA and protein synthesis and interact with intracellular targets such as DNA, RNA, and enzymes [1,7,47] (Figure 2d–f). Although AMPs are often described as being effective against a single class of microorganisms, a single AMP may exhibit activity against diverse microorganism groups through different mechanisms and can have lethal effects on various cell types via same mechanisms. The most effective AMPs are often those that disrupt membrane integrity, leading to rapid cell death through leakage of cellular contents. Approximately 33% of bacterial proteins are membrane-associated and functional in critical processes such as nutrient transport, respiration, proton motive force, and ATP production [1]. AMPs can also be effective by causing these proteins to lose their function or become dysfunctional without leading to damage to the cell membrane [1].

The selectivity of AMPs depends on the composition of the membrane content. Since bacterial membranes contain anionic lipids such as cardiolipin and phosphatidylglycerol, they strongly interact with cationic peptides. In contrast, AMPs weakly interact with eukaryotic membranes due to their composition involving neutral zwitterionic phospholipids such as sphingomyelin and phosphatidylcholine. Furthermore, cholesterol found in mammalian membranes increases membrane stability and prevents the entry of AMPs into the membrane [1,43,48,49]. Therefore, mammalian cells are less affected by AMPs than microbial cells. Ergosterol contributes to fungal cell membrane stability similarly to cholesterol; however, fungal membranes have a more anionic composition compared to mammalian membranes. This composition increases the electrostatic interactions between AMPs and the membrane. Therefore, despite the stabilizing effect of ergosterol, AMPs can still strongly interact with fungal membranes [8,50].

The permeability of cell walls varies between Gram-positive and Gram-negative bacteria, owing to the distinct composition of their cell walls. These differences determine the ability of AMPs to cross these barriers. The cytoplasmic membrane of Gram-positive bacteria is surrounded by a 40–60 nm thick peptidoglycan layer containing negatively charged teichoic acid. The cell wall has small, nano-sized pores that allow AMPs to reach the cytoplasmic membrane by diffusion. In Gram-negative bacteria, the presence of a lipopolysaccharide outer membrane (LPS), rich in negatively charged phosphate groups, in conjunction with peptidoglycan is a strong barrier to AMPs to reaching the cytoplasmic membrane. The LPS layer forms an electrostatic network by interacting with divalent cations. The network also acts as a barrier for hydrophobic molecules such as antibiotics. This double membrane structure makes it more difficult for AMPs to attach to the cell membrane and degrade the cell wall compared to Gram-positive bacteria [2,44].

AMPs are now used as alternative innovative compounds to antibiotics in combating microorganisms that have developed multidrug resistance. Unlike conventional antibiotics, which typically target specific pathways or proteins, many AMPs exert their effects through multiple action mechanisms, making it difficult for pathogens to develop resistance to these antimicrobials [2,51]. Their specificity and selectivity can be enhanced by changing the biophysical properties. Additionally, new anti-infective agents can be developed from them by optimizing other desired biological characteristics such as low toxicity, increased stability, or host cell compatibility [1,52,53]. It has been demonstrated that AMPs possess various biological activities and therapeutic properties, including cytotoxic effects against cancer cells, antimicrobial effects against various microorganisms, and anti-inflammatory, immunomodulatory, and endotoxin neutralizing activities. These comprehensive features have enabled AMPs to be evaluated as suitable agents for pharmacological applications [54]. In addition, AMPs have high utilization potential in the food industry as food additives and packaging materials [55,56]. They are also used for protection and control against diseases in the aquaculture industry and livestock farming [57,58], as well as in the fight against pathogens for plant protection [8,54,59]. Among the diverse AMPs, plant-derived peptides have attracted considerable attention due to their broad-spectrum activities. In particular, the snakin family of plant-based AMPs is a promising candidate for use in the agriculture, food and pharmaceutical industries due to its antimicrobial activity against both phytopathogenic and clinical species.

This review specifically focuses on the snakin AMP family because of its broad-spectrum antimicrobial activity and structural uniqueness. Their biochemical and molecular properties are discussed, along with potential uses in biotechnological applications. It also presents updated versions of various database sources. Unlike previous reports, this review compiles snakin peptides with antimicrobial activity obtained from screening of the UniProt database at both the transcript and protein levels. Considering the need for novel strategies to combat antimicrobial resistance, snakins have become prominent, promising non-antibiotic agents capable of disrupting resistance mechanisms through multi-targeted modes of action. Therefore, they also have significant potential as natural tools for practical applications. By compiling the current knowledge, this review offers a broad perspective to researchers on the subject.

## 2. Plant AMPs

Plant AMPs are peptides that function in defense mechanisms against biotic and abiotic stress responses and also play roles in various physiological and developmental processes [60]. They have molecular weights ranging from 2 to 10 kDa, are generally positively charged at physiological pH, and can be isolated from all vegetative and generative plant organs [54,61,62]. These peptides are generally rich in cysteine residues (4–12 cysteine residues). Disulfide bonds formed between cysteine residues give them high chemical, thermal, and proteolytic stability [63]. Purothionin was the first identified plant AMP with antimicrobial activity, which was isolated from wheat (*Triticum aestivum*) [64]. To date, a large number of plant-derived AMP groups have been identified, classified, and characterized, and a number of them have been utilized after purification. The amino acid sequence similarity, presence of cysteine motif and tertiary structure formation are used as basic criteria for their classification [62,63]. They are classified into defensins, thionins, lipid transfer proteins, cyclotides, hevein-like peptides, and snakins based on amino acid sequence similarities [39,65]. The observation that certain plant AMPs exhibit inhibitory activity against phytopathogenic as well as species that pose a threat to public health has attracted considerable interest regarding their potential agricultural and therapeutic applications [39,62].

## 3. Snakins

Snakins are a group of relatively small (~7 kDa) plant AMPs that are typically rich in cysteine residues. Most members of this group contain 12 conserved cysteine residues in the C-terminus, called the GASA (gibberellic acid-stimulated in *Arabidopsis*) domain, which is characteristic of the GASA peptide family. This cysteine-rich motif plays a crucial role in the structural stability and functional activity of the snakin family. Snakins also contain a putative N-terminal signal peptide and a variable region in the middle of their sequences. Due to their conserved amino acid sequence homology, snakins are also designated as the snakin/GASA family [62,66,67]. Snakin-1 (SN1, also known as StSN1, UniProt: Q948Z4) (https://www.uniprot.org/uniprotkb/Q948Z4/entry (Accessed 27 March 2026)), snakin-2 (SN2, also known as StSN2, Uniprot: Q93X17) (https://www.uniprot.org/uniprotkb/Q93X17/entry (Accessed 27 March 2026)) and snakin-Z peptides were identified by characterization methods carried out after protein isolation from plants, while others were identified by genomic analysis. Moreover, high-throughput in silico analyses have facilitated the determination of novel snakin peptides with potential antimicrobial activity against bacterial, fungal, and viral pathogens [67].

Snakin/GASA peptides are encoded by a multigene family, and their regulation are associated with phytohormones such as gibberellin (also known as gibberellic acid), salicylic acid and abscisic acid [60,67]. In this context, approximately 445 genes have been identified from 33 plant species [68]. The number of Snakin/GASA family genes varies among plant species, ranging from 5 to 37 (*Petunia hybrida* 5, *Oryza sativa* 9, *Zea mays* 10, *Arabidopsis thaliana* 15, and *S. tuberosum* 18, *Triticum aestivum* and *Glycine max* 37 genes). Their homologs are not found in algae, green algae and animals. However, bacterial species including *Escherichia coli*, *Klebsiella pneumoniae*, *Nitriliruptoraceae bacterium*, *Acinetobacter baumannii*, *Soehngenia saccharolytica*, *Glycocaulis profundi*, and *Staphylococcus warneri* have been shown to have these gene homologs [67]. The role of segmental duplications in the formation of the GASA gene family has been demonstrated by synteny analyses. In addition, cis-element analysis showed that cis-regulatory elements found in the promoter region of these genes can be induced by abiotic factors [69].

The StSN1 (UniProt: Q948Z4) and StSN2 (Uniprot: Q93X17) peptides, isolated from potato (*Solanum tuberosum*) tubers, are the most studied members of the snakin/GASA family [67,70,71]. The first identified snakin peptide was StSN1. It was named snakin due to its similar sequence motifs to snake venom [62,67,70]. StSN1 and StSN2 have 38% amino acid sequence similarity. However, both AMPs exhibit similar antimicrobial activity against bacteria and phytopathogenic fungi [71,72]. In vitro studies with StSN1 and StSN2 show that, unlike other plant-derived AMPs, they do not interact with artificial lipid membranes. Although they rapidly aggregated in the membranes of both Gram-positive and Gram-negative bacteria, this property did not correlate with their inhibitory activity [70,71]. Kuddus et al. [73] reported that recombinant StSN1 is a membrane-active AMP that disrupts both extracellular and plasma membranes of *E. coli*; Shwaiki et al. [74] showed that synthetic StSN1 causes permeabilization of the cell membrane of spoilage yeasts. SN2 from *Solanum lycopersicum* has been reported to induce the formation of non-specific pores in the membranes of pathogenic microorganisms [75,76]. In addition to targeting membrane components of pathogens, it has been reported that snakins may also exhibit their antimicrobial properties by binding to microbial DNA and/or RNA [60,66,77]. Despite these insights, the precise mode of action of snakin peptides remains unclear and requires further investigation.

SN1 and SN2 homologs have been reported from diverse plant species recently (Table 2). The antimicrobial activity of all snakin peptides against various parasites, phytopathogenic and clinical microorganisms were demonstrated by in vitro, in planta and in silico studies (Table 2). Importantly, although the antimicrobial activity of AMPs against multidrug-resistant (MDR) microorganisms has been demonstrated, there are only a limited number of studies investigating the effects of snakins on MDR isolates. In this context, PdSN1 has been shown to exhibit moderate inhibitory activity against a *Staphylococcus aureus* strain (ATCC 6538P) (1.80 μM, 56.3% inhibition) [77], which was previously described as moderately resistant to penicillin, although this resistance could not be confirmed using susceptibility testing based on Clinical Laboratory Standard Institute guidelines. In addition, a possible strong interaction of SN1 with penicillin-binding protein 2a of methicillin-resistant *S. aureus* was determined through an in silico study [78].

The gene encoding StSN1 is expressed in different tissues of potato except in roots, stolons, and leaves, and peptide synthesis is not affected by biotic and abiotic stress factors [70,71]. Silencing of this gene affected cell division, primary metabolism, and cell wall composition and caused changes in potato size, leaf size, and leaf morphology. These data suggest that SN1 may have a functional role in plant growth and development in addition to its role in the defense system [96]. The gene responsible for StSN2 production is continuously expressed in all tissues of the potato except in roots, stolons, and sepals. Its expression level is also locally up-regulated in case of infection and/or damage [71]. Transcriptomic studies with the homolog of this gene in tomato (*S. lycopersicum*) showed similar expression patterns. The fact that SN2 has been demonstrated to increase tomato tolerance against the pathogenic bacterium *C. michiganensis* suggests its active role in the defense system against pathogenic microorganisms [75,88]. Sequences coding both SN1 and SN2 have been used to confer disease resistance in various plants through transgenic technologies. Within this context, these sequences have been successfully transferred into economically important plants such as tomato [88], potato [83,84,87], wheat [80,85], rice [82], lettuce [86], and alfalfa [92] using different gene transfer methods, including biolistic and *Agrobacterium*-mediated technologies, under various promoter control systems, and have been demonstrated to be powerful biotechnological tools against bacterial, fungal, viral, and nematode-derived diseases.

Snakin-Z peptide is a 31-amino acid AMP isolated from the fruit of the jujube plant (*Z. jujuba*). It is used as an anticancer, antipyretic, analgesic, analgesic, appetite stimulant, anticoagulant and tonic. The identified snakin-Z amino acid sequences lack 12 cysteine residues that are highly conserved in the snakin/GASA family [67,97,98]. Although snakin-Z was reported to have 39.39% amino acid similarity with SN2 peptide, amino acid alignments performed with the CLUSTALW program showed that snakin-Z has 70.9677% homology with SN2, 38.7097% homology with SN1, and 35.2273% similarity with SN1 and SN2. The antimicrobial activity of snakin-Z against various bacterial and fungal species has been demonstrated [95] (Table 2). In addition, Teker et al. [98] show the antibacterial effect on *S. aureus* clinic isolate (ATCC 25923) of heterologously expressed glutathione S-transferase (GST)-fused snakin-Z.

In recent years, computational methods have been used to investigate the activity of AMPs and their interactions with microbial membrane models, and have yielded valuable insights into activity prediction and mechanisms of action. In this context, Kumar et al. [99] predicted the binding mechanisms of snakin-Z to model membranes belonging to bacteria, fungi, and human red blood cells (RBCs) employing computational modeling approaches. They demonstrated that this AMP binds more strongly to *B. subtilis* and *C. albicans* membranes than to membranes of *E. coli* and RBCs, primarily due to differences in lipid composition. The binding stability of snakin-Z was associated with its amphipathic structure and specific residues, particularly Arg28, key contributors to the interaction with *B. subtilis*, *C. albicans*, and *E. coli* membrane models. It has been found that the high cholesterol content in the membranes of RBCs helps to preserve membrane integrity and reduces peptide-induced disruption; these findings are consistent with the low hemolytic activity observed in experiments. All these outputs provide mechanistic insights into snakin-Z’s selectivity and support its potential as a safe and effective plant-derived AMP.

Producing snakin/GASA peptide using recombinant DNA technology has eliminated the limitations associated with obtaining this peptide from natural sources, such as low yield and high extraction costs [98]. However, due to their small size, cationic nature, and cysteine-rich composition, snakin peptides may pose challenges in prokaryotic expression systems such as *E. coli*, including toxicity to host cells, insolubility, and improper folding [100]. These issues can be overcome by employing strategies such as codon optimization, production via fusion with carrier proteins (e.g., thioredoxin, GST), the use of specialized host strains, and tandem multimeric gene expression [101]. Baculovirus-infected insect cell systems provide an alternative, suitable platform for the expression of this peptide [100]. The recombinant peptides produced from these systems serve not only as tools for structural and functional studies but also for practical applications in agriculture, food systems, and health.

Transgenic approaches involving overexpression or silencing technologies have been applied either to enhance or reduce resistance to various phytopathogens in agriculture [67,84]. Broad-spectrum antimicrobial activities and non-specific pore-forming abilities of snakins suggest their potential as a natural preservative to prevent microbial spoilage in the food and cosmetics industries [100]. The low hemolytic activity of SN1, SN2, and snakin-Z against human RBCs indicates their potential as therapeutics [60,95,102]. Furthermore, the report that snakin-Z exhibited inhibitory activity against the antioxidant enzymes acetylcholinesterase and butyrylcholinesterase, which are associated with neurodegenerative disorders, supports its potential for use in the treatment of Alzheimer’s disease [103].

Although it has been determined that SN2 is sensitive to high salt concentrations and digestive enzymes, and its use as a systemic drug in the human body is therefore limited, this AMP remains a promising candidate for topical applications, such as in lotions or wound dressings. Additionally, it has been demonstrated that SN2 facilitates the entry of antibiotics into pathogenic cells when used in combination. In this case, SN2 may lead to reduced antibiotic use in the treatment of infections and, as a result, may have the potential to limit the development of antimicrobial resistance in pathogenic microorganisms [76].

## 4. Conclusions and Future Perspectives

The snakin family is a promising family of plant AMPs for both pharmaceutical and agricultural applications. These peptides are involved in a wide range of plant physiological processes and play a crucial role in plant innate immunity. Their ability to combat various pathogenic microorganisms, including those exhibiting multi-drug resistance, positions them as effective alternatives to traditional antibiotics. Their biological characteristics render them attractive biotechnological targets across diverse industrial applications. This comprehensive analysis emphasizes the rich diversity and functional capabilities of AMPs, paving the way for innovative solutions in both plant health management and combating antibiotic resistance in clinical settings. Further extensive structural and functional investigations focused on the snakin family may lead to the development of novel antimicrobial agents that increase plant resistance and contribute to sustainable agricultural practices. Continued research and investment in AMPs will be essential to unlock their full capacity for mitigating microbial threats in both human and environmental contexts.

## Figures and Tables

**Table 1:** List of AMP databases and their contents.

Database	Database Content	Total Number of Entries	Reference
AMPDB v1 ^1^	AMPs	59,122	[9]
AntiTbPdb ^2^	Experimentally verified anti-tubercular or anti-mycobacterial peptides	1010	[10]
APD6 ^3^	Natural AMPs, predicted and synthetic AMPs	6309	[11]
AVPpred ^4^	Peptides with antiviral activity	1245	[12]
BaAMPs ^5^	AMPs specifically tested against microbial biofilms	Not available	[13]
BACTIBASE ^6^	Bacteriocins produced by both Gram-positive and Gram-negative bacteria	230	[14]
BAGEL4 ^7^	BAGEL4 is a web server that enables users to identify and visualize gene clusters in prokaryotic DNA involved in the biosynthesis of ribosomally synthesized and post translationally modified Peptides (RiPPs) and (unmodified) bacteriocins	Not available	[15]
BioPepDB ^8^	Food-derived bioactive peptides	4807	[16]
BIOPEP-UWM ^9^	Biologically active peptides derived from food, sensory peptides and amino acids, proteins	5684	[17]
CAMPR4 ^10^	Natural and synthetic AMPs	24,243	[18]
CancerPPD2 ^11^	Experimentally verified anticancer peptides and proteins	6521	[19]
CPPsite 2.0 ^12^	Cell penetrating peptides	1700	[20]
DADP ^13^	Anuran defense peptides	2571	[21]
DBAASP v3 ^14^	Experimentally tested ribosomal, nonribosomal, and synthetic peptides that show antimicrobial activity as monomers, multimers, and multi-peptides	>15,700	[22]
dbAMP 3.0 ^15^	Experimentally verified AMPs and putative AMPs	35,518	[23]
DFBP ^16^	Food-derived bioactive peptides	6276	[24]
DRAMP 4.0 ^17^	Entries are categorized as general entries, patent entries, clinical entries, stapled entries, stability data and expanded entries	30,260	[25]
DRAVP ^18^	Antiviral peptides and proteins	5688	[26]
FermFooDb ^19^	Biologically active peptides derived from fermented food	2205	[27]
Hemolytik2 ^20^	Experimentally validated hemolytic and non-hemolytic peptides	13,215	[28]
HIPdb ^21^	Experimentally verified HIV inhibiting peptides	981	[29]
HORDB ^22^	Peptide hormones	7390	[30]
InverPep ^23^	Experimentally validated AMPs from invertebrates	702	[31]
LAMP2 ^24^	Natural and synthetic AMPs	23,253	[32]
MBPDB ^25^	Bioactive peptides derived from milk proteins	691	[33]
NeuroPep 2.0 ^26^	Neuropeptides	11,417	[34]
PEPLab ^27^	Food-derived peptides	2784	[35]
Peptaibols ^28^	Peptides known as peptaibols	Not available	[36]
PepTherDia ^29^	Approved peptide drugs and diagnostic agents	105	[37]
PlantPepDB ^30^	Plant peptides	3848	[38]
PhytAMP ^31^	Plant AMPs	271	[39]
THPdb2 ^32^	Approved and/or investigational therapeutic peptides	6385	[40]
YADAMP ^33^	AMPs	2133	[41]

^1^ https://bblserver.org.in/ampdb/ (Accessed 9 May 2026); ^2^ http://webs.iiitd.edu.in/raghava/antitbpdb/ (Accessed 9 May 2026); ^3^ https://aps.unmc.edu/AP/ (Accessed 9 May 2026); ^4^ http://crdd.osdd.net/servers/avppred/index.html (Accessed 1 February 2024); ^5^ https://www.baamps.it (Accessed 9 May 2026); ^6^ https://bactibase.hammamilab.org/ (Accessed 9 May 2026); ^7^ http://bagel4.molgenrug.nl/ (Accessed 9 May 2026); ^8^ https://bis.zju.edu.cn/biopepdbr/index.php?p=help (Accessed 9 May 2026); ^9^ https://biochemia.uwm.edu.pl/biopep/start_biopep.php (Accessed 9 May 2026); ^10^ https://camp.bicnirrh.res.in/ (Accessed 9 May 2026); ^11^ https://webs.iiitd.edu.in/raghava/cancerppd2/api/rest.html (Accessed 9 May 2026); ^12^ http://crdd.osdd.net/raghava/cppsite/ (Accessed 1 February 2024); ^13^ http://split4.pmfst.hr/dadp/ (Accessed 1 February 2024); ^14^ https://dbaasp.org (Accessed 9 May 2026); ^15^ https://ycclab.cuhk.edu.cn/dbAMP/ (Accessed 9 May 2026); ^16^ http://www.cqudfbp.net/ (Accessed 24 January 2026); ^17^ https://dramp.cpu-bioinfor.org/ (Accessed 9 May 2026); ^18^ https://dravp.cpu-bioinfor.org/ (Accessed 9 May 2026); ^19^ https://webs.iiitd.edu.in/raghava/fermfoodb (Accessed 9 May 2026); ^20^ http://webs.iiitd.edu.in/raghava/hemolytik2/ (Accessed 9 May 2026); ^21^ http://crdd.osdd.net/servers/hipdb/ (Accessed 1 February 2024); ^22^ http://hordb.cpu-bioinfor.org (Accessed 9 May 2026); ^23^ https://ciencias.medellin.unal.edu.co/gruposdeinvestigacion/prospeccionydisenobiomoleculas/InverPep/public/home_en (Accessed 9 May 2026); ^24^ http://biotechlab.fudan.edu.cn/database/lamp/index.php (Accessed 1 February 2024); ^25^ https://mbpdb.nws.oregonstate.edu/peptiline/ (Accessed 9 May 2026); ^26^ http://www.isyslab.info/NeuroPepV2/home.jsp (Accessed 9 May 2026); ^27^ https://www.pep-lab.info/ (Accessed 9 May 2026); ^28^ https://www.cryst.bbk.ac.uk/peptaibol (Accessed 29 March 2026); ^29^ https://peptherdia.herokuapp.com/ (Accessed 9 May 2026); ^30^ http://14.139.61.8/PlantPepDB/ (Accessed 9 May 2026); ^31^ https://phytamp.hammamilab.org/ (Accessed 9 May 2026); ^32^ https://webs.iiitd.edu.in/raghava/thpdb2/ (Accessed 9 May 2026); ^33^ http://www.yadamp.unisa.it (Accessed 9 May 2026).

**Table 2:** Antimicrobial properties of snakins in various plants. * EC_50_ (50% effective concentration); IC_50_: 50% inhibitory concentration; MIC: Minimum inhibitory concentration; MMC: Minimum microbicidal concentration; ** represents phytopathogenic species; *** represents the clinical isolates.

Plant Species/Protein	Expression Strategy/ Experimental Approach(es)	Target Pathogens	Type of Effect *	Reference
*Solanum tuberosum* (Potato)/SN1 (also known as StSN1 or GSL1)	Natural isolation from potato tubers/in vitro	*Clavibacter michiganensis* subsp. *Sepedonicus* **	Antibacterial activity (EC_50_ < 10 μM); synergistic with potato defensin	[70]
		*Botrytis cinerea* **	Antifungal activity (EC_50_ = 3 μM); additive effect with potato defensin	
		*Fusarium solani* **	Antifungal activity (EC_50_ < 10 μM)	
		*Bipolaris maydis* **	Antifungal activity (EC_50_ < 10 μM)	
		*Colletotrichum lagenarium* **	Antifungal activity (EC_50_ < 10 μM)	
		*Aspergillus flavus* **	No antifungal activity observed	
		*Ralstonia solanacearum* **	No antibacterial activity observed	
	Recombinant expression in *E. coli*/in vitro	*C. michiganensis* subsp. *sepedonicus* AS1 **	Antibacterial activity (IC_50_: 1.50–8 μM)	[79]
		*C. coccoides* **	Antifungal activity (IC_50_: 5–14 μM)	
		*B. cinerea* **	Antifungal activity (IC_50_: 5–14 μM)	
		*Pseudomonas syringae* pv. *Syringae* 61 **	Weak antibacterial activity alone; strong synergistic effect when combined with potato defensin	
		*P. syringae* pv. *tabaci* 11528 Race 0 **	Weak antibacterial activity alone; additive effect when combined with potato defensin	
	Overexpression in transgenic wheat plant/in planta	*Gaeumannomyces graminis* **	Increased resistance in transgenic wheat	[80]
		*B. sorokiniana* **		
	Recombinant expression in *Pichia pastoris*/in vitro	*Listeria monocytogenes* ATCC 19111 **	Antibacterial activity (MMC: 20 µM)	[73]
		*Salmonella enterica Serovar Typhimurium* ATCC 13311 ***	Antibacterial activity (MMC: 5–10 µM)	
		*E. coli* ML35 ATCC 43827	Antibacterial activity (MMC: 5–10 µM)	
		*P. pastoris* GS115ATCC 20864	Antifungal activity (MFC: 10 µM)	
		*Candida parapsilosis* ATCC 22019 ***	Antifungal activity (MFC: 5 µM)	
		*F. oxysporum* f. sp. *lycopersici* JCM 12575 **	Completely inhibited spore germination (60 µM)	
	Overexpression in transgenic *Poncirus trifoliate* (citrus)/in planta	*Xanthomonas citri* **	Significant reduction in citrus canker disease severity	[81]
	Synthetic peptide/in vitro	*Zygosaccharomyces bailli* Sa 1403	Fungicidal activity (MIC = 100–200 μg/mL)	[74]
		*Debaromyces hansenii* CBS2334	Fungistatic activity (MIC = 200–400 μg/mL)	
		*Z. rouxii* ATCC14679	No antifungal activity observed	
		*Saccharomyces cerevisiae*	No antifungal activity observed	
		*Kluyveromyces lactis* ATCC56498	No antifungal activity observed	
	Overexpression in transgenic *Oryza sativa* (rice)/in planta, in vitro	*Rhizoctonia solani* **	Antifungal activity by the crude protein from transgenic leaves; enhanced protection against the sheath blight disease	[82]
	Overexpression in transgenic potato/in planta	*Pectobacterium atrosepticum* **	Increased resistance to blackleg disease in transgenic potato	[83]
*Solanum chacoense* (Potato)/SN1	Overexpression in transgenic potato/in planta	*R. solani* AG 3 **	Enhanced resistance, reduced disease symptoms and higher survival rates in transgenic potatoes	[84]
		*Erwinia carotovora* subsp. *carotovora* **	Enhanced resistance; reduced lesion size and symptom severity in transgenic potatoes	
	Overexpression in transgenic wheat plant/in planta	*R. solani* **	Enhanced resistance in transgenic wheat	[85]
		*E. carotovora* **		
	Overexpression in transgenic *Lactuca sativa* (lettuce)/in planta, in vitro	*R. solani* **	Antifungal activity by extracts of transgenic lettuce; enhanced tolerance in transgenic lettuce plants	[86]
		*Sclerotinia sclerotiorum* **	Enhanced tolerance in transgenic lettuce plants	
*S. tuberosum*/StSN2 (also known as SN2 and GSL2)	Natural isolation from potato tubers/in vitro	*C. michiganensis* subsp. *sepedonicus*	Antibacterial activity (EC_50_: 1 μM)	[71]
		*R. solanacearum*	No antibacterial activity observed	
		*E. chrysanthemi*	No antibacterial activity observed	
		*Rhizobium meliloti*	Antibacterial activity (EC_50_: 8 μM)	
		*B. cinerea*	Antifungal activity (EC_50_: 2 μM)	
		*F. solani*	Antifungal activity (EC_50_: 3 μM)	
		*F. culmorum*	Antifungal activity (EC_50_: 2 μM)	
		*A. flavus*	Antifungal activity (EC_50_: 20 μM)	
		*B. maydis*	Antifungal activity (EC_50_: 20 μM)	
		*C. lagenarium*	Antifungal activity (EC_50_: 10 μM)	
		*C. graminicola*	Antifungal activity (EC_50_: 10 μM)	
		*F. oxysporum* f. sp. *lycopersici*	Antifungal activity (EC_50_: 20 μM)	
		*F. oxysporum* f. sp. *conglutinans*	Antifungal activity (EC_50_: 10 μM)	
		*Plectosphaerella cucumerina*	Antifungal activity (EC_50_: 10 μM)	
	Overexpression in transgenic potato/in planta	*P. atrosepticum* (formerly *E. carotovora* subsp. atroseptica) **	Increased resistance in transgenic potato	[87]
*S. lycopersicum* (Tomato)/SN2	Recombinant expression in *E. coli*/in vitro	*E. coli* DH5α	Antibacterial activity (MIC: 4.25 μM)	[75,76]
		*Agrobacterium tumefaciens*	Antibacterial activity (MIC: 1.06 μM)	
		*Micrococcus luteus*	Antibacterial activity (MIC: 0.26 μM)	
		*S. cohnii*	Antibacterial activity (MIC: 1.06 μM)	
		*P. pastoris*	Antifungal activity (MIC: 8.49 μM)	
		*F. solani* **	Antifungal activity (MIC: 4.25 μM)	
		*B. subtilis*	Antibacterial activity (MIC: 2.12 μM)	
		*S. cerevisiae*	Antifungal activity (MIC: 4.25 μM)	
	Overexpression in transgenic tomato/in planta	*C. michiganensis* subsp. *michiganensis* **	Enhanced tolerance in transgenic tomato	[88]
	Gene silencing in *Nicotiana benthamiana*/in planta	*C. michiganensis* **	Increased host susceptibility to bacterial pathogens	[89]
*Allium cepa* (Onion)/Snakin 1–7	Bioinformatics analysis/in silico	Human, animal and plant pathogens	Potential antimicrobial activity	[90]
*Capsicum annuum* (Pepper)/CaSnakin	Recombinant expression in *E. coli*/in vitro	Free-living nematodes (*Caenorhabditis elegans* N2)	Antimicrobial activity	[91]
		Root-knot nematodes (*Meloidogyne* spp.)	Antimicrobial activity	
*Medicago sativa* (Alfalfa)/MsSN1	Recombinant expression in *E. coli*/in vitro	*A. tumefaciens* LBA4404 **	Inhibits bacterial growth	[92]
		*Phoma medicaginis* var. medicaginis CBS 316.90 **	Inhibits fungal spore germination	
	Overexpression in transgenic alfalfa/in planta, in vitro	*P. medicaginis* CT1 **	Significantly lower percentage of diseased leaflets in transgenic alfalfa plant	
		*C. trifolii* CT2 **		
*Panax notoginseng* (Chinese notoginseng)/PnSN1	Recombinant expression in *E. coli*/in vitro	*F. solani* **	Inhibits mycelial growth and spore germination of the fungal pathogen at concentrations of 4, 8 and 16 μg	[93]
		*F. oxysporum* **	Inhibits mycelial growth of fungal pathogen	
		*F. verticillioides* (Sacc.) Nirenb. **		
		*Botryosphaeria dothidea* **		
	Overexpression in transgenic tobacco/in planta	*F. solani* **	Increased the resistance	
*Peltophorum dubium* (Fabaceae)/PdSN1	Recombinant expression in *E. coli*/in vitro	*C. albicans* CCMG13 ***	Antifungal activity (IC_50_: 1.20 μM; 65.50% inhibition at 1.80 μM)	[77]
		*A. niger* CCMG17 ***	Antifungal activity (IC_50_: 1.40 μM; 56.70% inhibition at 1.80 μM)	
		*B. cinerea* CCMG14 g	Antifungal activity (IC_50_: 0.40 μM; 53.60% inhibition at 1.80 μM	
		*Alternaria alternata* CBS916.96	Antifungal activity (IC_50_: 0.40 μM; 58% inhibition at 1.80 μM	
		*Streptomyces scabies* DSM41658 **	Antibacterial activity (IC_50_: 0.30 μM; 99.70% inhibition at 1.80 μM)	
		*S. aureus* ATCC6538P ***	Antibacterial activity; 60% inhibition at 1.80 μM of PdSN1	
		*C. michiganensis* ssp. *Michiganensis* MAI1008	Antibacterial activity (IC_50_: 1.70 μM; 56.30% inhibition at 1.80 μM)	
		*E. coli* CCMG50	No antibacterial activity at 1.80 μM	
		*X. vesicatoria* MAI2020	No antibacterial activity at 1.80 μM	
		*Penicillium expansum* CCMG14s	No antifungal activity observed at 1.80 μM	
*Persea americana* var. *drymifolia* (Avocado)/PaSN	Heterologous expression in bovine endothelial cells (BVE-E6E7)/in vitro	*E. coli* 0111 ***	100 mg/mL Pa inhibits the viability of *E. coli* by 90.70%	[94]
		*S. aureus* 27543 ***	100 mg/mL inhibits the viability of *S. aureus* by 89.80%	
*Zizyphus jujuba* (Chinese date)/Snakin-Z	Natural isolation from potato tubers/in vitro	*E. coli* PTCC2433	Antibacterial activity (MIC: 13.60 mg/mL)	[95]
		*S. aureus* PTCC1442	Antibacterial activity (MIC: 28.80 mg/mL)	
		*Klebsiella pneumonia* PTCC4231	Antibacterial activity (MIC: 14.10 mg/mL)	
		*Phomopsis azadirachtae* PTCC5027	Antibacterial activity (MIC: 7.65 mg/mL)	
		*Pythium ultimum* PTCC5021	Antibacterial activity (MIC: 8.36 mg/mL)	
		*A. niger* ***	Antifungal activity (MIC: 9.30 mg/mL)	
		*C. albicans* PTCC4236	Antifungal activity (MIC: 8.23 mg/mL)	
		*B. subtilis* ***	Antibacterial activity (MIC: 24.20 mg/mL)	

## Data Availability

No new data were created or analyzed in this study. Data sharing is not applicable to this article.

## Abbreviations

The following abbreviations are used in this manuscript:

AMPs	Antimicrobial peptides
APD	Antimicrobial Peptide Database
ATP	Adenosine Triphosphate
DNA	Deoxyribonucleic Acid
EC_50_	Effective concentration 50
GASA	Gibberellic Acid-Stimulated in Arabidopsis
GST	Glutathione S-transferase
IC_50_	50% inhibitory concentration
LPS	Lipopolysaccharide
MDR	Multidrug-resistant
MIC	Minimum inhibitory concentration
MMC	Minimum microbicidal concentration
RBCs	Red Blood Cells
RNA	Ribonucleic Acid
SN1	Snakin-1
SN2	Snakin-2
